# Using a Caesarean Section Classification System based on characteristics of the population as a way of monitoring obstetric practice

**DOI:** 10.1186/1742-4755-7-13

**Published:** 2010-06-26

**Authors:** Maria L Costa, Jose G Cecatti, João P Souza, Helaine M Milanez, Metin A Gülmezoglu

**Affiliations:** 1 Department of Obstetrics and Gynecology, School of Medical Sciences, University of Campinas (UNICAMP), Brazil; 2 UNDP/UNFPA/WHO/World Bank Special Programme of Research, Development and Research Training in Human Reproduction, Department of Reproductive Health and Research, HRP/WHO, Geneva, Switzerland

## Abstract

**Objective:**

to compare the distribution of caesarean rates in the Robson's 10 groups classification in order to see if any change occurred after the implementation of an audit and feedback intervention. *Design: *cross sectional, before and after an audit and feedback study. *Setting*: a university hospital in Brazil.

**Methods:**

clinical records of all births during two three months-periods were evaluated. Each case of CS was classified into one of ten mutually exclusive categories according to obstetric characteristics. The proportion of CS in each group was compared in both periods.

**Results:**

total number of deliveries and the high rate of CS were similar in both periods. Group 3 (multiparous excluding previous CS, single, cephalic, ≥ 37 weeks, spontaneous labour) accounted for the largest proportion of deliveries, 28.5 and 26.8% in both periods. Group 1 (nulliparous, single, cephalic, ≥ 37 weeks, spontaneous labour) was the second largest one, while Group 5 (previous caesarean section, single, cephalic, and ≥ 37 weeks) was the third but the largest contributor to CS, accounting for 16.6 and 14.9% among all deliveries in both periods. Groups 2 (nulliparous, single, cephalic, ≥ 37 weeks, induction or CS before labour) and 4 (multiparous excluding previous CS, single, cephalic, ≥ 37 weeks, induction or CS before labour) were less prevalent, however had higher rates of CS. Only in Group 10 (All single, cephalic, ≤ 36 weeks, including previous CS), there was a significant decrease of CS rate from 70.5 to 42.6% between periods.

**Conclusion:**

Robson's classification did not identify any significant change in the pattern of CS rates with the audit and feedback process, but showed to be useful for comparing trends among similar obstetric populations.

## Background

Discussion on caesarean section rates, efforts to prevent its continuous increase and the possibility to allow patients choose their delivery route has been an important topic throughout the world [1].

The World Health Organization stated in 1985 that no region should exceed rates higher than 10-15 percent of caesarean sections [2]. However, those goals seem no longer to be greatly achievable, both in developed and developing countries. In fact, the highest increases in Caesarean section rates occurred especially in Latin American countries during the seventies and eighties. Data available for Brazil show that the overall rate of caesarean section for the country as whole was 30% of all deliveries, reaching as high as 50% of deliveries in certain provinces around two decades ago, while currently data show that the overall rate is 33% and even more for private institutions (51%) among a sample of Latin American countries [3-5]. Although the mean world total caesarean sections is estimated around 15% as recommended, there are enormous regional differences, for instance 3.5% in Africa and 40.5% in Eastern Asia, currently one of the places with the highest rates [6].

Caesarean section rates seem to vary by country, states within a country, type of facility (private versus public) and the level and type of caregiver [7]. That has led to numerous studies of interventions with the purpose to understand and reduce the caesarean section rate [8-10]. Audit and feedback and multifaceted strategies are considered useful interventions for reducing caesarean section rates [11]. However, to improve the effectiveness of these interventions, it would be useful to fully understand the determinants of caesarean section in each setting.

The use of a classification system applicable internationally and designed to allow both short and long term analysis of determinants and implications of caesarean sections would be necessary [1]. In order to be a successful classification, the information collected should be useful, carefully defined, accurately collected, timely and available. A classification system strictly based on the obstetric characteristics of the population, with mutually exclusive and totally inclusive categories has been developed. The Robson system is simple to understand and implement, the groups are considered to be clinically relevant and meets the requirements outlined. The 10 groups are intended only to give an initial overview of caesarean section rates that can be compared with rates either in different units or in the same unit over time. Following the initial comparison, each of the groups may need to be studied further to determine the reasons for the differences [1]. This classification has already been showed to be useful for monitoring CS rates and their components, calling the attention for the higher prevalent groups [12].

The present study aims to test the implementation of the Robson's Classification for CS based on obstetric categories at a referral university hospital in Brazil with a high rate of caesarean section, in the context of an audit and feedback study performed to try to improve obstetrical care comparing two three-months periods. The objective is to compare the groups' distribution of caesarean rates in order to see if any change occurred after the implementation of an audit and feedback intervention.

## Material and methods

An Audit and Feedback study design was performed at the University of Campinas, Brazil, in which six evidence-based practices were monitored. The methodological aspects and main results of this study are fully described elsewhere [13]. Basically it was decided to audit six standard procedures with strong evidence favoring or contra-indicating its use in obstetric practice: selective episiotomy, continuous electronic fetal heart rate monitoring during uncomplicated labour of low-risk pregnancies, antibiotic prophylaxis for caesarean section, active management of third stage of labour, routine induction of labour at 41 weeks for uncomplicated pregnancies, and continuous support for women during childbirth. At least three of these included practices could have an impact on caesarean section rates: not to perform continuous electronic fetal monitoring (EFM) during labour for low risk pregnant women [14], to induce labour in otherwise low-risk pregnancies after completing 41 weeks of gestational age [15] and continuous support during labour and childbirth by a companion [16]. A prospective medical audit of the CS characteristics was performed, evaluating the clinical records of all deliveries during two three months periods: April to June of 2007 and November of 2007 to January of 2008, separated by a fourth month intervention period when information and reinforcement on the use of those practices were given to all obstetric staff of the institution, including assistant professors, medical officers, residents in obstetrics and gynecology, medical students and nurses. They received information on the current utilization rates of those practices, plus refreshing and reinforcement on their updated evidences through classes with presentations, seminars and workshops, face to face talks, ward rounds, documents and having the Reproductive Health Library (RHL) from WHO freely available in the computer network of the institution. The study was approved by the local Institutional Review Board and received financial support from FAPESP (Foundation for Support to Research of the State of Sao Paulo). There was no informed consent for each woman, because data was not collected in an individual base.

The data was compiled to fill Robson's Classification of CS with the final objective of identifying the groups of women contributing most to the caesarean section rate. Robson's Classification of CS [1] defines 10 groups, according to different combinations of the women's obstetric record, category of pregnancy (single pregnancy: cephalic, breech or oblique; multiple pregnancy), the presence of previous uterine scar, the course of labour and delivery (spontaneous labour, induced labour, caesarean section before labour) and the gestational age.

Based on the known mean prevalence of caesarean section rate in the institution of around 45%, and assuming that a minimum clinically significant desired reduction would be of 8%, with a confidence of 95% and a power of 80%, at least 617 cases would be necessary for each group. Taking into account the mean number of deliveries per month of around 200-220, it was considered that three months would be necessary for the pre and post intervention periods. According to the characteristics of each case delivering in the periods studied, the data collection form was filled including the case in one of the Robson's group. Daily, during all the period of the study, three forms were completed, one for the total deliveries of that day, one for vaginal deliveries and the last for caesarean deliveries. These forms were pooled first weekly, then monthly and finally for the three-month period before and after intervention. For data analysis, the distribution of cases among all 10 groups of Robson's classification for total births, for caesarean section deliveries and the contribution of each group to the overall CS rates were compared between pre and post intervention periods, with differences assessed with Chi-square or Exact of Fisher tests.

## Results

The total number of deliveries occurring in the first period was 664 and 628 in the second period after intervention. The proportion of CS was similar and high in both periods (45.5% and 43.3% in the pre and post intervention periods respectively). Relating every caesarean section to the total number of women in each group according to Robson's Classification, there was a similar distribution of the 10 groups upon both periods considered (Table 1).

**Table 1:** Number of Caesarean Sections over total number of women in each group by Robson's classification according to pre and post intervention period

Groups	Number of CS over total number of women in each group	
	
	Pre intervention	Post intervention
1. Nulliparous, single cephalic, ≥ 37 weeks, in spontaneous labour	41/170	36/143
2. Nulliparous, single cephalic, ≥ 37 weeks, induced or CS before labour	26/40	30/47
3. Multiparous (excluding prev. CS), single cephalic, ≥ 37 weeks, in spontaneous labour	44/189	35/169
4. Multiparous (excluding prev. CS), single cephalic, ≥ 37 weeks, induced or CS before labour	15/31	23/45
5. Previous CS, single cephalic, ≥ 37 weeks	110/152	94/134
6. All nulliparous breeches	9/9	8/8
7. All multiparous breeches (including prev. CS)	13/13	12/12
8. All multiple pregnancies (including prev. CS)	10/13	7/12
9. All abnormal lies (including prev. CS)	3/3	4/4
10. All single cephalic, ≤ 36 weeks (including prev. CS)	31/44	23/54
Total*	302/664	272/628
	**45.5%**	**43.3%**

p = 0.4663

The majority of women in both time periods were in group 3, of multiparous women without a previous uterine scar, with a single pregnancy at term, in spontaneous labour, cephalic presentation, accounting for respectively 28.5% and 26.9% during the first and second three-month period. The second major was group 1, nulliparous women with a single cephalic pregnancy, above 37 weeks in spontaneous labour, with percentages of 25.6% and 22.7% respectively. The third was group 5, all multiparous women, with at least one previous uterine scar and a single cephalic pregnancy above 37 weeks, with 22.9% and 21.3% respectively. Another important group to consider was number 10, all women with a single cephalic pregnancy below 37 weeks gestation, including women with previous scars, with percentages of 6.6% and 8.6% respectively. The variations between both periods were not statistically significant (Table 2).

**Table 2:** Relative size of each group by Robson's classification according to pre and post intervention period

Groups	Relative size of groups %	
	
	Pre intervention	Post intervention
1. Nulliparous, single cephalic, ≥ 37 weeks, in spontaneous labour	**25.6**	**22.7**
	(170/664)	(143/628)
		
2. Nulliparous, single cephalic, ≥ 37 weeks, induced or CS before labour	**6.0**	**7.5**
	(40/664)	(47/628)
		
3. Multiparous (excluding prev. CS), single cephalic, ≥ 37 weeks, in spontaneous labour	**28.5**	**26.9**
	(189/664)	(169/628)
		
4. Multiparous (excluding prev. CS), single cephalic, ≥ 37 weeks, induced or CS before labour	**4.7**	**7.2**
	(31/664)	(45/628)
		
5. Previous CS, single cephalic, ≥ 37 weeks	**22.9**	**21.3**
	(152/664)	(134/628)
		
6. All nulliparous breeches	**1.3**	**1.3**
	(9/664)	(8/628)
		
7. All multiparous breeches (including prev. CS)	**2.0**	**1.9**
	(13/664)	(12/628)
		
8. All multiple pregnancies (including prev. CS)	**2.0**	**1.9**
	(13/664)	(12/628)
		
9. All abnormal lies (including prev. CS)	**0.5**	**0.6**
	(3/664)	(4/628)
		
10. All single cephalic, ≤ 36 weeks (including prev. CS)	**6.6**	**8.6**
	(44/664)	(54/628)

χ^2 ^= 8.213 p = 0.51

Regarding caesarean section rate in each group (Table 3), during both periods considered there were a 100% prevalence of caesarean sections in breech and other abnormal lies pregnancies (groups 6, 7 and 9). The lower rates of caesarean sections were seen in groups 1 and 3, with percentages ranging from around 20 to 24%. As expected, group 5, with at least one previous caesarean section, showed high rates of caesarean section, from 72.4% in pre intervention to 70.1% in post intervention period. All these differences again were not statistically significant. However, for Group 10, basically all preterm births, there was a significant decrease of caesarean section rates from 70.5 to 42.6% in both periods.

**Table 3:** Caesarean section rate in each group by Robson's classification according to pre and post intervention period

Groups	CS rate in each group %		
	
	Pre intervention	Post intervention	p-value*
1. Nulliparous, single cephalic, ≥ 37 weeks, in spontaneous labour	**24.1**	**25.2**	0.9810
	(41/170)	(36/143)	
			
2. Nulliparous, single cephalic, ≥ 37 weeks, induced or CS before labour	**65.0**	**63.8**	0.9116
	(26/40)	(30/47)	
			
3. Multiparous (excluding prev. CS), single cephalic, ≥ 37 weeks, in spontaneous labour	**23.3**	**20.7**	0.4941
	(44/189)	(35/169)	
			
4. Multiparous (excluding prev. CS), single cephalic, ≥ 37 weeks, induced or CS before labour	**48.4**	**51.1**	0.8154
	(15/31)	(23/45)	
			
5. Previous CS, single cephalic, ≥ 37 weeks	**72.4**	**70.1**	0.7770
	(110/152)	(94/134)	
			
6. All nulliparous breeches	**100.0**	**100.0**	-
	(9/9)	(8/8)	
			
7. All multiparous breeches (including prev. CS)	**100.0**	**100.0**	-
	(13/13)	(12/12)	
			
8. All multiple pregnancies (including prev. CS)	**76.9**	**58.3**	0.4110
	(10/13)	(7/12)	
			
9. All abnormal lies (including prev. CS)	**100.0**	**100.0**	-
	(3/3)	(4/4)	
			
10. All single cephalic, ≤ 36 weeks (including prev. CS)	**70.5**	**42.6**	0.0058
	(31/44)	(23/54)	

*p-value according to Chi-square or Exact of Fisher tests

Considering the contribution of each group to the overall caesarean section rate in both periods (Table 4), the highest rates were for group 5, with all multiparous women, with at least one previous uterine scar and a single cephalic pregnancy at greater than or equal to 37 weeks gestation, with percentages of 16.6% and 14.9% respectively for pre and post intervention periods. They were then followed by groups 3 and 1. Group 10 showed a decrease from 4.7 in the pre intervention period to 3.7% in the post intervention period. However, overall these variations were also not statistically significant.

**Table 4:** Contribution of each group by Robson's classification to the overall Caesarean section rate according to pre and post intervention period

Groups	Contribution of each group to the overall CS rate %	
	
	Pre intervention	Post intervention
1. Nulliparous, single cephalic, ≥ 37 weeks, in spontaneous labour	**6.2**	**5.7**
	(41/664)	(36/628)
		
2. Nulliparous, single cephalic, ≥ 37 weeks, induced or CS before labour	**3.9**	**4.8**
	(26/664)	(30/628)
		
3. Multiparous (excluding prev. CS), single cephalic, ≥ 37 weeks, in spontaneous labour	**6.6**	**5.6**
	(44/664)	(35/628)
		
4. Multiparous (excluding prev. CS), single cephalic, ≥ 37 weeks, induced or CS before labour	**2.3**	**3.7**
	(15/664)	(23/628)
		
5. Previous CS, single cephalic, ≥ 37 weeks	**16.6**	**14.9**
	(110/664)	(94/628)
		
6. All nulliparous breeches	**1.4**	**1.3**
	(9/664)	(8/628)
		
7. All multiparous breeches (including prev. CS)	**1.9**	**1.9**
	(13/664)	(12/628)
		
8. All multiple pregnancies (including prev. CS)	**1.5**	**1.1**
	(10/664)	(7/628)
		
9. All abnormal lies (including prev. CS)	**0.4**	**0.6**
	(3/664)	(4/628)
		
10. All single cephalic, ≤ 36 weeks (including prev. CS)	**4.7**	**3.7**
	(31/664)	(23/628)

χ^2 ^= 5.3035 p = 0.8071

## Discussion

The results of the current study showed that Groups 3 and 1 were the two largest groups of women admitted for delivery and also those with the lowest proportion of caesarean sections. However, due to their large size, both accounted for considerable total number of caesarean sections. These results are in accordance to the two other studies already published on the same classification [1,12]. In addition, this study showed no significant variation in the proportional contribution of each group of Robson's classification to the caesarean section rates comparatively between pre and post intervention periods, unless for group 10 of preterm births where a decrease in caesarean section occurred between both periods.

Recent studies have shown an increase in groups 2 and 4, which are becoming larger and larger contributors to the overall caesarean section rates due to an enlargement in induction rates as well as caesarean sections before labour. The present study shows a relatively low participation of group 2 and 4 of around 6-7% among all deliveries. However, there were high rates of caesarean sections within each one of these two considered groups. This makes to arise an appropriate discussion on both, indication and methods for induction of labour and also elective caesarean sections before labour, probably a great proportion without a clear indication at all [5].

Probably the most markedly difference is the current higher proportion of group 5, women with at least one previous caesarean section scar among all deliveries, what represents a good reflection of the current Brazilian situation, two to three times higher than those already reported in Australia and Ireland [1,12]. Group 5 is a heterogeneous group, since it includes women with one or more scars, some with previous vaginal deliveries, and also women who either went into spontaneous labour, were induced or were delivered by caesarean section before labour [1]. This group is very important because it is the biggest contributor to the overall caesarean section rate, with percentages around 15%. Within its group, the total of caesarean section rate is also very high, accounting for around 71%. Therefore, definitely if any programme should be implemented to specifically reduce caesarean section rates, this group should be highlighted and focused with special intervention procedures, perhaps better addressing the alternatives for obtaining VBAC, mainly among those women with only one previous caesarean scar [17,18].

Group 10 should be carefully studied, since tertiary referral centers are expected to have elevated rates of preterm deliveries, due to the management of high risk pregnancies. The size of this group accounted for the fourth larger number of total deliveries, with around 7.5% of them. Within its group, the rate of caesarean section was also very high. While these results are in accordance with those already available in terms of proportional participation of the group among all deliveries, the current caesarean section rates are much higher [1,12]. It is interesting to note that this group was the only where a significant decrease of caesarean section occurred from pre to post intervention periods, from 70.5% to 42.6%. We had no explanation at all for such a result. This should be the subject of further in depth analysis.

The current study has identifiable limitations, the design as a before and after evaluation, the time period considered was diminutive and its initial purpose was to focus on the effect of Audit and Feedback [19,20] as an implementation method to study six obstetric practices underwritten by evidence-based medicine. The intervention was not initially planned to reduce caesarean rates, however, three of the practices selected could be able to reflect on a decrease of caesareans: continuous support during labour and childbirth by a companion, do not perform continuous electronic fetal monitoring (EFM) during labour of low risk pregnant women and to induce labour in low risk pregnancies when reaching 41 weeks of gestational age. However, only one of those (continuous support) presented a significant effect after the intervention period, which had no strength to demonstrate any change on caesarean rates.

To our knowledge, Robson's classification has already been used in five other contexts [1,12,21-23], four in developed countries (Ireland, Australia, United States and Sweden) and one in developing country (Chile), to categorize and understand the reality of each setting. In addition, a secondary analysis of the WHO Global Survey on Maternal and Perinatal Health in Latin America, analyzing data prospectively collected for almost one hundred thousand deliveries in eight countries from the region, identified Group 5 of women with a term singleton cephalic pregnancy with a previous caesarean section as the largest contributor to overall caesarean section rate, reaching almost 27% of all caesarean sections [24]. Nevertheless, the ten group classification was never considered to compare results and periods after an intervention, and this is exactly the innovative aspect of this study.

To further evaluate possible interventions that could be able to influence caesarean section rates, it would be important to study each of the groups initially considered as significant and if necessary, subdivide them. The present study showed extremely high rates of caesarean sections, with an overall percentage of around 45% in both periods evaluated. There would be possible interventions in almost all the groups considered to be addressed in further studies.

Robson's classification of caesarean sections can be easily performed to provide the framework for evaluating caesarean section rates and their implications. In the present study, applied to both periods did not identify any significant change in the pattern of caesarean section rates that could be attributable to the implementation of the evidence based obstetrical practices focused in the study. But the implementation of such classification system allowed comparing similar obstetric populations, what seems to be advisable for monitoring trends in caesarean section rates, especially when there is a policy trying to reduce high rates.

## Abbreviations

CS: caesarean section; EFM: electronic fetal monitoring; FAPESP: Foundation for Support to Research of the State of Sao Paulo; RHL: Reproductive Health Library; VBAC: vaginal birth after caesarean; WHO: World Health Organization.

## Disclosure of interests

The authors declare that they have no competing interests.

## Authors' contributions

MG had the original idea for the study. MLC wrote the first version of the proposal with orientation from MG and JGC. JGC and HMM got the grant for implementation of the study. MLC was responsible for data collection. JPS had the idea for the current analysis. MLC, HMM, JPS and JGC were responsible for data analysis. MLC, JPS and JGC wrote the first draft of the paper and then all the others gave important inputs and suggestions for interpretation and improvement of the manuscript. All authors have read the final version of the article and agreed with it.
